# Developments in Carbohydrate-Based Metzincin Inhibitors

**DOI:** 10.3390/ph13110376

**Published:** 2020-11-10

**Authors:** Doretta Cuffaro, Elisa Nuti, Felicia D’Andrea, Armando Rossello

**Affiliations:** Department of Pharmacy, University of Pisa, via Bonanno 6, 56126 Pisa, Italy; doretta.cuffaro@farm.unipi.it (D.C.); felicia.dandrea@farm.unipi.it (F.D.); armando.rossello@farm.unipi.it (A.R.)

**Keywords:** carbohydrates, glycoconjugates, MMPs, ADAMs, iminosugars, metzincin inhibitors

## Abstract

Matrix metalloproteinases (MMPs) and A disintegrin and Metalloproteinase (ADAMs) are zinc-dependent endopeptidases belonging to the metzincin superfamily. Upregulation of metzincin activity is a major feature in many serious pathologies such as cancer, inflammations, and infections. In the last decades, many classes of small molecules have been developed directed to inhibit these enzymes. The principal shortcomings that have hindered clinical development of metzincin inhibitors are low selectivity for the target enzyme, poor water solubility, and long-term toxicity. Over the last 15 years, a novel approach to improve solubility and bioavailability of metzincin inhibitors has been the synthesis of carbohydrate-based compounds. This strategy consists of linking a hydrophilic sugar moiety to an aromatic lipophilic scaffold. This review aims to describe the development of sugar-based and azasugar-based derivatives as metzincin inhibitors and their activity in several pathological models.

## 1. Introduction

### 1.1. Metzincins

Tissue remodeling is a crucial process in various pathological and physiological events in living organisms. In general, protein degradation during tissue turnover is regulated by a multitude of proteases, among which metzincins play an important role. Metzincin superfamily includes metalloproteinases which share a similar catalytic site constituted by a zinc ion, a zinc binding consensus motif, and a specific conserved methionine residue [1]. In the active form of the enzyme, the zinc ion is disposed in a highly conserved motif, tetrahedrally complexed with three histidines, and with a fourth coordination place available for the substrate and/or water. The consensus motif (HEXXHXXG/NXXH/D) is followed by a structurally conserved methionine which participates in the structural integrity of the catalytic domain [2]. Metzincins are classified in five different subfamilies on the basis of structural similarities: the matrixins (matrix metalloproteinases, MMPs), the serralysins (large bacterial proteinases), the astacins, the pappalysins and the adamalysins (ADAMs (A Disintegrin And Metalloproteinases), and ADAMTSs (ADAMs with thrombospondin motifs) [3].

### 1.2. Matrix Metalloproteinases

**Figure 1:**
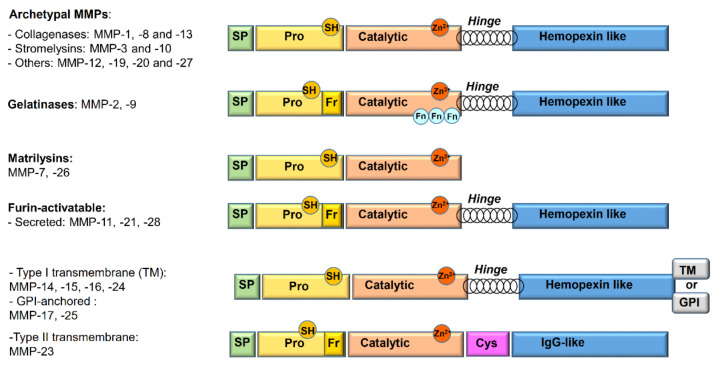
Schematic overview of the matrix metalloproteinase (MMP) domains and family member classification. SP: signal peptide; Pro: propeptide; TM: transmembrane domain; GPI: glycosylphosphatidylinositol; Cys: cysteine array; Fn: fibronectin repeat; Fr: furin-cleavage site; SH: thiol group.

Matrix metalloproteinases (MMPs), or matrixins, constitute a family of 24 zinc-dependent endopeptidases homogeneous for structure, function, and localization which are critical for the degradation of the extracellular matrix (ECM) [4]. In addition to their functions as tissue-remodeling enzymes, MMPs are also involved in the selective cleavage of many non-ECM targets, such as cytokines, cell surface receptors, chemokines, and cell–cell adhesion molecules [3]. The widely used classification of MMPs (Figure 1) is based on substrate specificity, sequence similarity, domain organization, and partially on their cellular organization [2,3]:

- Archetypal MMPs have a similar structure and are divided into:
  - Collagenases (MMP-1, MMP-8, MMP-13): specifically cleave the collagen triple helix. Moreover, their collagenolytic activity, due to a cooperation between the hemopexin and the catalytic domains, acts on a various number of ECM and non-ECM molecules.
  - Stromelysins (MMP-3, MMP-10): structurally similar to collagenases, show wide substrate specificity but are unable to degrade native collagen.
  - Others (MMP-12, MMP-19, MMP-20, and MMP-27): not classified in the previous categories. Metalloelastase (MMP-12) is mainly expressed in macrophages and digests elastin and other proteins.
- Gelatinases (MMP-2 or Gelatinase A and MMP-9 or Gelatinase B): owing to the presence of three fibronectin type II repeats inside the catalytic domain, they readily digest type IV collagen, gelatin, and a number of ECM molecules including laminin, fibronectin, and aggrecan core proteins.
- Matrilysins (MMP-7 and MMP-26): characterized by the lack of the hinge region and of hemopexin domain.
- Furin-activatable MMPs:
  - Secreted (MMP-11, MMP-21, and MMP-28): activated by furin-like proteases before secretion.
  - Membrane-type MMPs (MT-MMPs): classified in type I transmembrane proteins (MMP-14, MMP-15, MMP-16, and MMP-24) and type II transmembrane protein (MMP-23).
  - Glycosylphosphatidylinositol (GPI)-anchored proteins (MMP-17 and MMP-25).

Currently, the 3D structures of a number of MMPs have been determined by X-ray crystallography and NMR methods [5,6,7,8]. From a structural point of view, the family of MMPs mostly shares a common three domain-based structure that consists of a pro-peptide domain (about 80 residues) at the amino-terminal, a central catalytic domain (about 160–170 residues), and a hemopexin domain at the carboxylic-terminal. The pro-peptide domain confers latency to the enzyme by occupying the active site zinc, making the catalytic domain inaccessible to substrates. The pro-domain contains a sequence PR**C**GXPD with a conserved cysteine, fundamental for the activation process. The *catalytic domain* is strictly conserved among MMPs and also shows similarity for all metalloproteinases, leading to difficulties in site-targeted selective inhibitor design. It presents a specific consensus motif (His-Glu-X-X-His-X-X-Gly-X-X-His) typical of all MMPs. In addition, two structural zinc ions and at least one calcium ion are included. A hinge domain (about 60–70 residues) connects the catalytic domain to the hemopexin one [9].

The catalytic mechanism starts with the enzyme activation due to proteolytic cleavage of the *N*-terminal pro-domain. The removal of the pro-peptide disrupts the interaction between the conserved cysteine and the zinc, allowing the thiol group to be replaced by a water molecule. This so called “cysteine-switch mechanism” enables the partially activated enzyme to further hydrolyze the pro-peptide, resulting in its removal [4].

**Figure 2:**
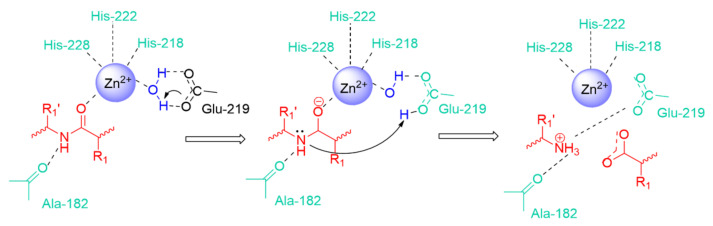
General representation of amide bond hydrolysis in the catalytic site of MMPs.

The substrate binding involves principally the zinc ion. In the active form of the enzyme, the zinc is tetrahedrally coordinated by three histidines (His 228, His 222, and His 218 in full MMP-3 numbering) and a water molecule. The glutamate residue, coordinating the water molecule, makes it a good nucleophile to attack the substrate scissile peptide bond (Figure 2).

#### MMP Catalytic Binding Site

**Figure 3:**
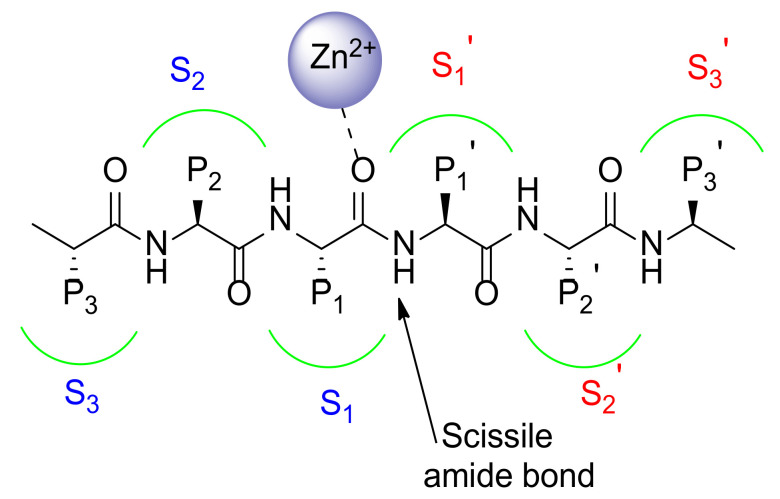
Schematic representation of MMP catalytic binding site.

The catalytic binding site as well as the substrates differ significantly among MMPs. Therefore, Schechter and Berger reported [10] a nomenclature system to identify amino acids in the substrate as well as the recognizing areas on the surface of the enzyme (Figure 3).

This generally accepted nomenclature identifies all of the subsites with S1, S2, and S3 for the sites on the left side of the zinc ion, while S1’, S2’, and S3’ are used to recognize the subsites on the right side of the zinc ion. The amino acid positions (P) of the substrate are countered from the scissile amide bond and have the same numbering to that of the subsites they occupy on the enzyme surface. Basically, the residues on the *N*-terminal side of the substrate are named P1, P2, and P3 whereas the residues on the *C*-terminal side are termed P1’, P2’, and P3’.

The MMP substrate binding cleft is mainly represented by a hydrophobic pocket, namely S1’ [11]. The S1’ pocket, also called the specificity pocket, confers the substrate-recognition varying among the different isoforms of MMPs in its aminoacidic sequence and depth. Although S2’ and S3’ pockets participate in substrate binding together with S1’, they are shallower pockets more solvent exposed than S1′. For these characteristics they result more difficult to target using synthetic inhibitors.

MMPs are classified on the basis of S1’ depth in shallow (MMP-1, MMP-7), medium (MMP-2, MMP-8, MMP-9), and deep (MMP-3, MMP-11, MMP-12, MMP-13, MMP-14) pocket MMPs. The S1’ pocket accommodates the side chain of the substrate that will become the new *N*-terminus [12].

### 1.3. ADAM Metalloproteinases

The vast majority of ADAMs are type I transmembrane proteins approximately constituted by 750 amino acids which belong to the metzincin superfamily [13,14].

ADAM structure is constituted by a metalloprotease domain, similarly to MMPs, and a unique disintegrin domain responsible for binding to integrins, which explains their name: ADAM (A Disintegrin and A Metalloproteinase). The main function of ADAMs is the cleavage of cell surface proteins among which are growth factors, cytokines, receptors, and adhesion molecules [15]. Their domain structure consists of a pro-domain, a metalloprotease domain, a disintegrin domain, a cysteine-rich domain, an EGF-like domain, a transmembrane domain, and a cytoplasmic tail. In the last years, 3D structures of the most important ADAMs have been published disclosing important structural information and specificities of the various domains [16,17,18,19].

The catalytic metalloproteinase domain is quite similar to the one of MMPs. The catalytic domain is divided into two subdomains, and the active site cleft is positioned between the two [2,20].

Similarly to MMPs, the groove between the two subdomains has six subsites (S3, S2, S1, S1’, S2’, S3’) determining specificity for particular amino acid sequences (P3, P2, P1, P1’, P2’, P3’) in the substrate. Particularly interesting are the subsites P1 and P1’, where the proteolytic cleavage occurs.

#### ADAM Ectodomain Shedding

The ADAMs have long been studied for their ability to cleave a large number of cell surface proteins, releasing a soluble fragment from its trans-membrane precursor. This mechanism is known as ectodomain shedding [21]. The cleaved transmembrane protein can be the substrate for cytoplasmic proteases or intramembrane proteolytic complexes, capable of releasing intracellular molecules responsible for gene transcription regulation. Alternatively, the ectodomain shedding, together with the protein internalization, can control the half-life of transmembrane proteins on the cell surface.

The ADAM mediated proteolysis is not only essential for providing extracellular signals, but also represents a prerequisite for intracellular signaling by regulated intramembrane proteolysis (RIP). RIP can be defined as a proteolytic cascade, initiated by an ectodomain sheddase action on a transmembrane protein which leads to intracellular release of soluble fragments [22].

### 1.4. Metzincin Inhibitors

Misregulation of the metzincin activity is a major feature in many serious pathologies [23]. In particular, it is well known that overexpression of these enzymes can lead to massive tissue degradation, dangerous for the promotion of cellular events such as tumor invasiveness [24] and inflammation spreading [25,26]. Moreover, an imbalanced expression of MMPs due to an inadequate regulation of TIMPs, is a hallmark of different bacterial, viral, or combined systemic infection such as sepsis [27,28]. Furthermore, recent findings about metzincin involvement in central nervous system (CNS) disorders, and in particular in Alzheimer’s disease (AD), make them modulators for promising therapeutic strategies [29,30,31]. The strong involvement of metzincins in these critical diseases has been repeatedly confirmed by gene-direct studies and animal models so that many resources have been invested in the last 30 years by pharmaceutical companies in the development of metzincin inhibitors [32].

A classical metzincin inhibitor consists of a “backbone” and a “zinc-binding group” (ZBG) [33]. The backbone is a classic drug-like structure designed to establish noncovalent bonds with the protein, such as hydrophobic and electrostatic interactions and hydrogen bonds. The zinc-binding group is a chelating group for the catalytic zinc atom that is also able to give hydrogen bonds with the enzyme. The hydroxamate group is the most used ZBG due to its excellent chelating properties [34]. The zinc chelation mediated by hydroxamates proceeds with a bidentate structure, in which each oxygen is located at an optimal distance to the catalytic zinc. Additionally, hydroxamate efficacy is potentiated by hydrogen bond formation between its heteroatoms and some residues conserved in all MMPs (the reason why this moiety suffers from poor selectivity).

**Figure 4:**
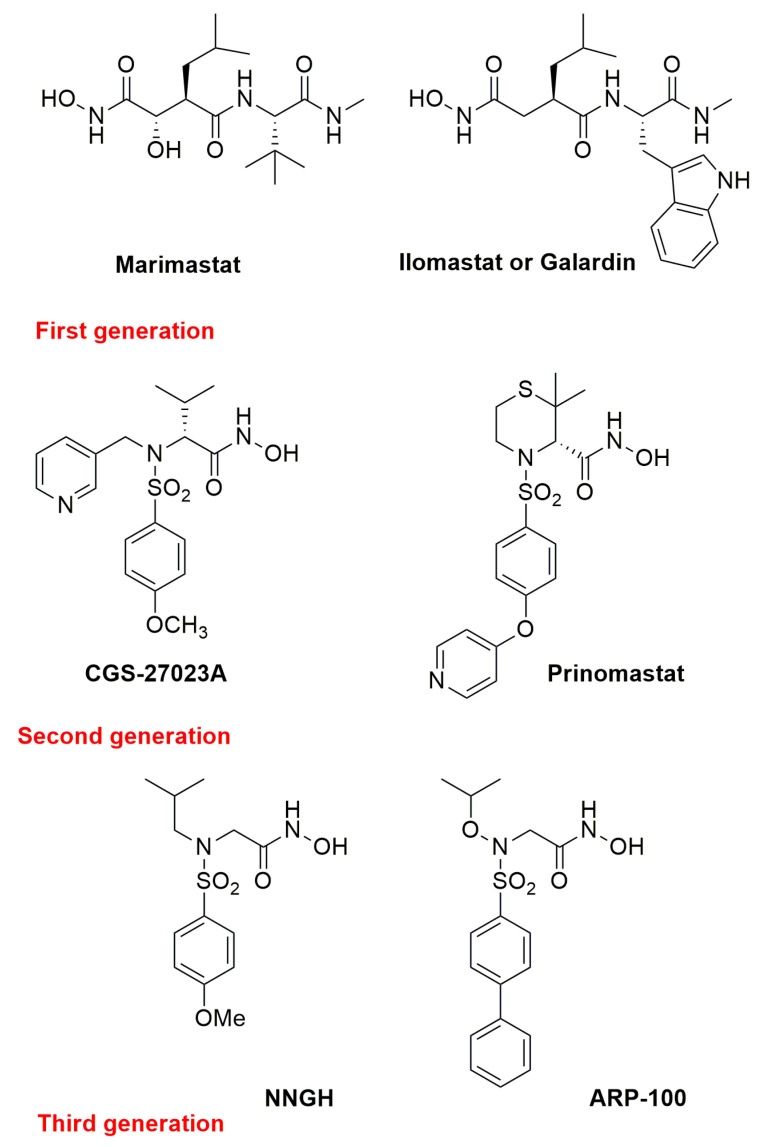
Chemical structure of the most representative zinc-chelating metzincin inhibitors.

Initially, three main generations of zinc-binding metzincin inhibitors were developed, exploiting the growing knowledge of these proteinases. Small molecules such as the peptidomimetic Marimastat, and Ilomastat (or Galardin) [35] (first generation, Figure 4), the sulfonamido-based CGS-27023A [36] and Prinomastat (second generation, Figure 4), NNGH [37] and ARP100 [38] (third generation, Figure 4), were identified as progenitors of many potent metzincin inhibitors [39]. Many MMP inhibitors showed high potency in preclinical studies but the selectivity goal was not achieved, and they eventually failed in clinical trials. The most important syndrome derived from the use of broad-spectrum metzincin inhibitors is a Musculoskeletal Syndrome (MSS) and has nowadays been ascribed to MMP-1 and MMP-14 inhibition [40].

In the last 15 years new strategies have been developed in order to selectively target MMPs such as using non-zinc binding inhibitors [41,42,43], or exosite-binding inhibitors [44,45,46].

The first synthetic ADAM inhibitors relied on Zn^2+^ binding groups to interact with the catalytic active site. One of the most studied ADAMs is ADAM17 (or TACE, TNF-α converting enzyme), a protease involved in the shedding of transmembrane protein ectodomains [47,48]. Recently, selective ADAM17 inhibitors able to target the catalytic binding site [49,50,51] or the enzyme exosites have been reported [52,53].

Although several small molecule MMP [54] or ADAM [55] inhibitors with high in vitro activity have been described, the principal obstacles that have hindered clinical development of metzincin inhibitors are the inadequate selectivity for the target enzyme, the poor water solubility and long-term toxicity. In fact, the majority of the metzincin inhibitors reported so far presented a hydrophobic structure which gave high affinity for the target enzyme due to hydrophobic interactions. However, the high lipophilicity of these scaffolds negatively affected their bioavailability, increasing the human serum albumin retention (HSA) [56] and hindering the target reach. A good water solubility would confer a good bioavailability in physiological media, representing a highly desirable property for drug-like compounds.

Therefore, in the last 15 years, glycoconjugation has been investigated as an approach to improve the hydrophilicity of metzincin inhibitors and thus increase their oral bioavailability, avoiding a detrimental effect on their inhibitory activity. In the next paragraph we will describe and fully detail all the carbohydrate-based inhibitors of MMPs and ADAMs reported in literature so far.

## 2. Carbohydrate-Based Metzincin Inhibitors

In this review carbohydrate-based metzincin inhibitors will be described and classified on the basis of the sugar moiety in sugar-based inhibitors and azasugar-based inhibitors.

### 2.1. Sugar-Based MMPIs

Nowadays, it is well known how a wide range of glycosylated derivatives such as glycoproteins or glycosylated natural products, are crucial for physiological [57] and pathological processes [58]. Particularly interesting is the protein-carbohydrate recognition, a phenomenon fundamental in many cellular mechanisms, such as migration, adhesion, cell-differentiation, tumor progression, infections (by viruses or bacteria), and immune response among others [59,60,61,62,63]. In the last years, the ubiquity but also the biological properties of glycoproteins or glycosylated natural products have driven research towards the synthesis of glycoconjugates for the development of drug candidates with anti-infectious, anti-inflammatory, anticancer, or vaccine activity [64,65,66].

Due to their polyhydroxylated nature, carbohydrates are often employed as biocompatible enhancers of the hydrophilic profile of water insoluble drugs. In the design and development of potential drugs, the study of physico-chemical properties (stability, lipophilicity, aqueous solubility, and bioavailability) is essential to obtain compounds with optimal potency and selectivity [67,68,69,70]. For this reason, the insertion of a sugar moiety could be crucial to improve the bioavailability profile but also to recognize specific cellular entities, thus increasing the activity of the parent non-glycoconjugated compound.

**Figure 5:**
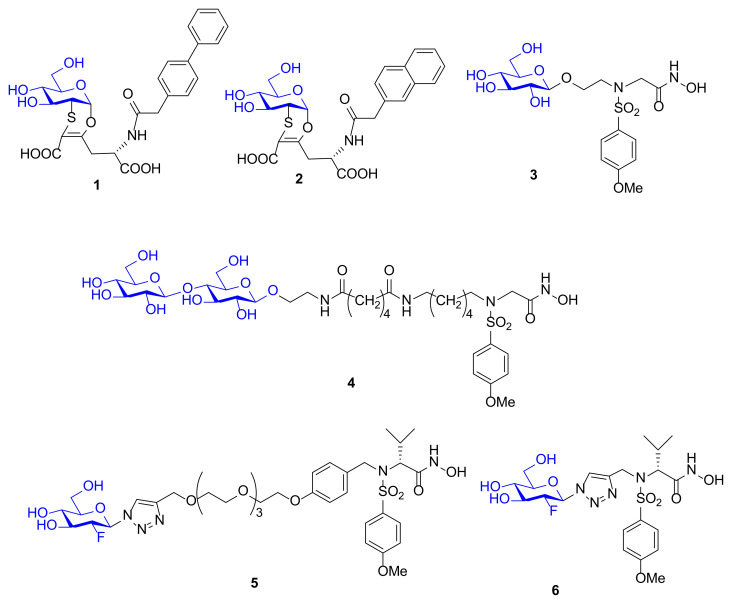
Chemical structure of glycoconjugated MMPIs **1–6**.

In the last years some water-soluble MMPIs have been reported in literature by different research groups [71]. The first two sugar-based MMPIs (**1** and **2**, Figure 5) were reported by Fragai et al., in 2005 [72]. These two compounds present a sulfur-containing constrained structure that can be synthesized in a diastereomerically pure α-*O*-glyco form through a totally chemo-, regio-, and stereoselective Diels–Alder reaction.

A virtual screening of various molecules has been carried out by docking analysis in MMP-12 catalytic domain. Compounds **1** and **2**, which are characterized by a side chain with a biphenyl group (**1**) or a naphthyl group (**2**) linked to the homoglutamic nitrogen and presenting as ZBG a carboxylic acid, afforded the best activity results. A NMR study on ligand–protein interactions was performed to confirm the in silico results. Binding of **1** and **2** to ^15^*N*-enriched MMP-12 catalytic domain was monitored by ^1^H,^15^N HSQC NMR spectroscopy and pointed out the lack of interactions between the carboxylic acid and the protein and confirmed the good interaction of the lipophilic moieties with the S1’ pocket of the enzyme. These compounds were poor MMP-12 inhibitors with IC_50_ in the high micromolar range but represent the first carbohydrate-based MMP-12 inhibitors reported in literature.

**Figure 6:**
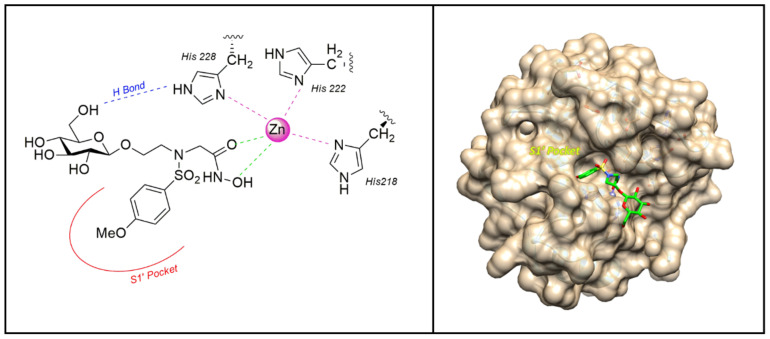
The binding mode of **3** [73] in the MMP-12 catalytic domain (PDB: 3N2U). Image generated with Chimera, version 1.13.

In 2006 the sulfonamido-based MMPI **3** (Figure 5) was published by Calderone et al. [73] as a more soluble derivative of NNGH [37] in which the isopropyl group was replaced by a glycosylated *N*-hydroxyethyl chain. The β-*O*-glucopyranoside derivative **3** showed a higher water solubility (>30 mM) compared to NNGH and presented a nanomolar activity (*K*_i_) for MMP-1 (286 nM), MMP-8 (9 nM), MMP-12 (14 nM), and MMP-13 (1.7 nM). The X-ray analysis of compound **3**-MMP-12 complex (Figure 6) evidenced a lack of significant participation in binding of the glucose ring. The MMP-12-compound **3** complex showed the classical coordination of the hydroxamate moiety with the catalytic zinc ion, the good fitting of the hydrophobic moiety in the S1’ pocket and the protrusion of the glucose portion out of the protein towards the solvent region. Although the glucose interaction with the protein could be marginal, it seemed to have an effective role on the selectivity profile for MMP-13 compared to other MMPs. Moreover, the interaction of compound **3** and NNGH with human serum albumin (HSA) was reported. While NNGH is a strong binder, inhibitor **3** did not appreciably interact with HSA, probably due to the glucose moiety.

Afterwards, the same research group reported the synthesis of a bifunctional MMPI, **4** (Figure 3), which was structurally related to compound **3**. The glucose unit of **3** was replaced by a lactose moiety and the spacer length was increased [74]. The lactose was chosen in order to bind Galectin-3 (Gal-3), a member of galectin family strictly involved in cancer progression. The presence of Gal-3 in tumor invasion area correlates with MMP upregulation. For this reason, a bifunctional architecture, which combines MMP inhibitory activity with galectin targeting, was adopted as design strategy. The inhibitory potency of compound **4**, tested on a set of MMPs, revealed that the lactose functionalization did not impair the activity of the ligand (*K*_i_s in the nanomolar range) and did not improve the selectivity. The ability of **4** to bind MMPs and Gal-3 simultaneously was demonstrated by a NMR study in MMP-12 catalytic domain. A ternary complex Gal-3-compound **4**-MMP-12 was formed in solution and the ligand **4** was able to gather Gal-3 and MMP-12 side by side.

In 2012, Hugenberg et al. [75] described a MMPI series of fluorinated triazole-substituted hydroxamates. Among them, two 2-fluoro-glucopyranoside derivatives, **5** and **6** (Figure 5), were synthesized by click chemistry reaction. These compounds differ only in the linker connection that is a triazole methyl chain in **6** and a triazole PEG chain in **5**. The conjugation was achieved through copper-catalyzed click chemistry reaction between 2-fluoro glucose and the alkynyl sulfonamide scaffold. The glucose derivatives reported high activity (*K*_i_ ranging picomolar for **5** and nanomolar for **6**) for MMP-2, MMP-8, MMP-9, and MMP-13 but no selectivity, and an excellent cLogD value. These compounds were designed in order to be radiolabeled as [^18^F] derivatives. The aim was to obtain suitable tools for in vivo imaging of activated MMPs with PET. Unfortunately, **5** and **6** were not chosen to be labeled for further studies, due to their poor selectivity.

**Figure 7:**
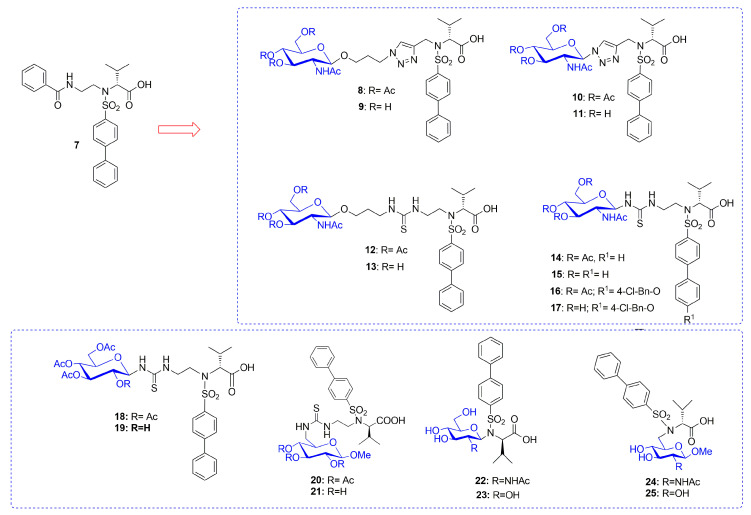
Chemical structure of sugar-based arylsulfonamido MMPIs **8–25** and the parent compound **7**.

**Figure 8:**
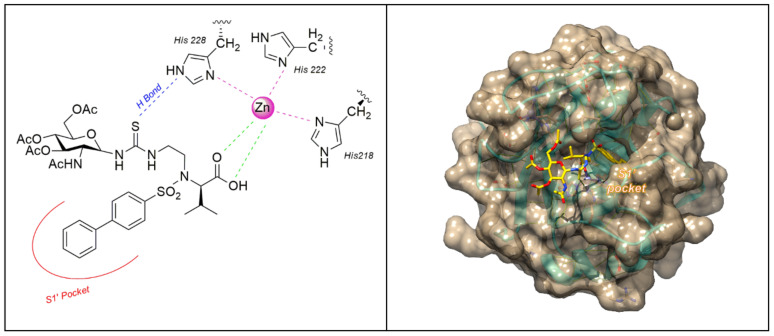
The binding mode of **14** [76] in MMP-12 catalytic domain (PDB: 5I0L). Image generated with Chimera, version 1.13.

Recently Rossello’s group reported two series of sugar-based arylsulfonamide carboxylates as MMP-12 inhibitors (**8–17** and **18–25**, Figure 7) [76,77]. Due to the long-time experience of this group into the design and synthesis of MMP-12 inhibitors [78,79,80,81], a previously published potent and selective arylsulfonamido MMP-12 inhibitor **7** [82] (Figure 7) was selected as starting compound. The structure of **7** was modified by replacing the aromatic ring on its sulfonamide nitrogen (P2’ position) with a β-*N-*acetyl-d-glucosamine (GlcNAc) moiety through insertion of a proper spacer. The aim was to improve the water solubility of **7**, avoiding a loss of activity for the target, by inserting a sugar moiety in a proper position of the arylsulfonamido scaffold. This choice was also supported by crystallographic studies of compound **7**-MMP-12 complex, which revealed the lack of interaction between the benzoamidoethyl group and the protein. The conjugation between GlcNAc and the MMPI-scaffold was obtained by introducing a thioureido group or a 1,4-triazole group. Glycoconjugates **8–17** (Figure 7) [76] were tested on human recombinant MMPs by a fluorometric assay, showing a stronger affinity for MMP-12 (IC_50_ in the nanomolar range) than for MMP-9 (IC_50_ in the micromolar range) for all derivatives. Compounds of the thioureido series, **12–17** (IC_50_ MMP-12 <40nM), were more active than the triazole analogues, **8–11** (IC_50_ MMP-12 < 75 nM). X-ray crystallographic analysis of the binding mode of **14** into MMP-12 catalytic domain (Figure 8) confirmed the better fitting for MMP-12 compared to MMP-9, and the higher affinity of thioureido derivatives with respect to triazole analogues. Furthermore, a physicochemical evaluation of thioureido derivatives **14–17** by cLogP in silico calculation and by UV-Vis solubility test, demonstrated the improvement in water solubility of all derivatives compared to parent compound **7**. The best glycoconjugated MMPI of this series was compound **15**, with a nanomolar activity for MMP-12 (IC_50_ 40nM), a very good selectivity profile over the other tested MMPs, and an improved hydrophilicity (water solubility > 5 mM and cLogP = 3.15).

On the basis of these encouraging results, in the following paper [77] the same group undertook the synthesis of new arylsulfonamido-based glycoconjugated MMP-12 inhibitors (**18–25** Figure 7) in order to perform a SAR analysis in this class of compounds. GlcNAc was replaced with glucose in order to improve intestinal absorption of the compounds targeting the glucose transporters (GLUT). In fact, glucose is actively absorbed by sodium dependent cotransporter 1 of intestinal epithelial cells [83]. Furthermore, the effect of the conjugation position on activity was analyzed by linking the hydrophobic scaffold in C-1 or C-6 position of the sugar through a Mitsunobu reaction or by inserting a thioureido spacer. All the compounds were tested by fluorometric assay and showed a nanomolar activity for MMP-12 except for some outliers. The best compounds were the glucose derivatives **18** and **19** with a nanomolar activity for the target and a good selectivity over MMP-9. Compounds **14**, **15**, **18**, and **19** resulted the best compounds of the two series and they were selected to evaluate their intestinal permeability using an ex-vivo everted gut sac model. Among the four glycoconjugates, GlcNAc-based compound **15** showed the best intestinal permeability and considering the similarity with glucose structure, it was supposed to cross the intestinal membrane by using the facilitative GLUT2 transporter.

Biological results for sugar-based MMPIs are summarized in Table 1.

**Figure 9:**
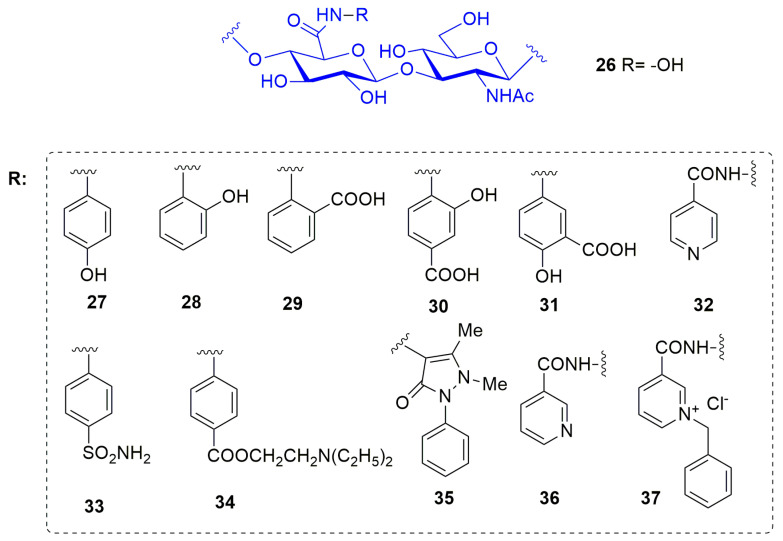
Chemical structure of hyaluronic acid-based MMP-2 inhibitors **26–37.**

In 2016 Ponedel’kina et al. [84] reported a water soluble hyaluronic acid (HA)-based hydroxamate and its derivatives conjugated with biologically active amines and hydrazides as MMP-2 inhibitors (**26–36**, Figure 9). HA is a natural non-immunogenic and biodegradable polysaccharide, consisting of alternated d-glucuronic acid and *N*-acetyl-d-glucosamine units widely applied in medicine as synovial fluid substitute and viscoelastic for ophthalmic surgery. In this study HA was functionalized as hydroxamic acid (**26**) and conjugated with biologically active amines and hydrazines through carbodiimide technique in water solution. The amines used are biologically relevant amines able to afford various glycoconjugates: *p*- and *o*-aminophenols **27** and **28**, anthranilic acid **29**, 4- and 5-aminosalicylic acids **30** and **31**, isonicotinic (Isoniazid) **32**, *p*-aminobenzenesulfonamide (Streptocide) **33**, *p*-aminobenzoic acid diethylaminoethyl ester (Procaine) **34**, 4-amino-2,3-dimethyl-1-phenyl-3-pyrazolin-5-one (4-aminoantipyrine) **35**, hydrazides **36** and benzyl pyridinium quaternary salt **37**. Moreover, they all contained groups able to coordinate the catalytic zinc ion or able to specifically interact with the binding site. The new compounds were tested by enzymatic assay on MMP-2 at 0.27–270 μM concentration range. Considering the percentage of inhibition, conjugates with *O*-aminophenol **28** and 4-aminoantipyrine **35** obtained 100% of inhibition at 0.27 μM concentration. 4-Aminosalicylic acid **30** was able to inhibit up to 92% at 270 μM in a dose dependent manner and other conjugates showed only 40–60% inhibitory effect at different concentrations.

### 2.2. Azasugar-Based Inhibitors

#### 2.2.1. Azasugar Biological Background

Azasugars or iminosugars, whether of natural or synthetic origin, are hydroxylated carbohydrate mimics, where a basic nitrogen substitutes the classic endocyclic oxygen [85]. This modification appears simple and not significant but provides important biological properties and also gives rise to many synthetic challenges [86,87,88,89,90,91,92]. Historically, iminosugars are known as inhibitors of glycosidases, enzymes involved in the glycosidic bond hydrolysis in different biological processes, such as intestinal digestion, post-translational processing of the sugar chain of glycoproteins, and lysosomal catabolism of glycoconjugates [93].

Despite being a structurally diversified class of molecules (polyhydroxylated piperidines and pyrrolidines and their derivatives), iminosugars attracted considerable interest due to their biomedical relevant activities. In fact, this class of compounds displayed different biological properties: as antiviral agents (against HIV-1, herpes simplex virus, bovine viral diarrhea virus (BVDV), and hepatitis C virus (HCV)), as antidiabetics, as agents for the treatment of lysosomal storage disorders such as Gaucher’s and Niemann–Pick type C, in immune modulation and as anticancer agents [94].

**Figure 10:**
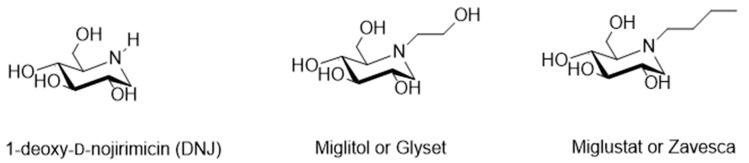
Chemical structure of DNJ, Miglitol and Zavesca.

Among these polyhydroxylated alkaloids, 1,5-dideoxy-1,5-imino-d-glucitol or 1-deoxy-d-nojirimycin [95] (DNJ, Figure 10) and its *N*-alkylated derivatives have prime importance. In fact, most of the commercially available azasugar drugs are based on DNJ structure, such as Glyset (*N*-hydroxyethyl-1-deoxy-d-nojirimycin, commonly known as Miglitol, Figure 10) [96,97,98], a well-known human alpha-glucosidase inhibitor used to treat type II diabetes and Zavesca (*N*-butyl-1-deoxy-d-nojirimycin commonly known as Miglustat, Figure 10) [99], which is an approved prominent inhibitor of glycosphingolipids for Gaucher disease. Recently DNJ has been studied also as MMPI, with not completely clear results. In 2010 Wang et al. [100] reported the DNJ effect on reducing B16F10 cell metastatic capability by suppressing the activity and the expression of MMP-2 and -9, but simultaneously imbalancing MMP-2 and TIMP-2 activity. Moreover, in 2013 the mulberry DNJ pleiotropic effect on the development of atherosclerosis was reported [101]. Mulberry DNJ is able to inhibit migration of A7r5 vascular smooth cells in a dose dependent manner and both the MMP-2 and MMP-9 activities in these cells. In this case the DNJ-inhibition of VSMC migration is a synergistic effect due to two different pathways: AMPK activation and the inhibition of F-actin activity. MMP-2 and MMP-9 inhibition is a key step in RhoB activation in the AMPK activation path.

#### 2.2.2. Azasugar-Based ADAM/MMP Inhibitors

**Figure 11:**
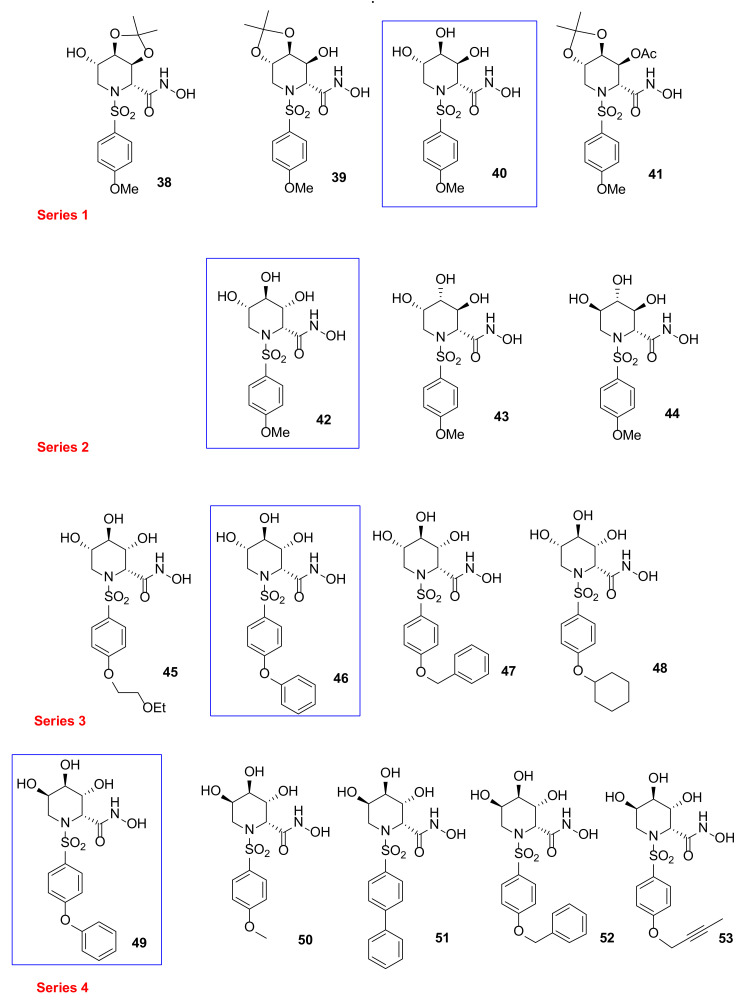
Chemical structure of azasugar-based MMP/ADAM inhibitors (Series 1–4).

Based on a classical MMP inhibitor scaffold, Moriyama et al., reported [102,103,104] the design, synthesis, and SAR study of azasugar-based MMP/ADAM inhibitors (**38–53**, Series 1–4 Figure 11). This class of compounds is based on the structure of known MMP inhibitors constituted by a sulfonamide scaffold as structural motif inserted in an azasugar having different configuration (l-*altro*, l-*ido*, l-*gluco*, l-*manno*, and l-*gulo*). The first series (Series 1 Figure 11) [102] was prepared to investigate the stereochemistry and the different protection of hydroxyl groups attached to a *p*-methoxysulfonamido scaffold. The inhibition data on MMP-1, MMP-3, MMP-9, and ADAM17 (also known as TACE: *tumor necrosis factor-α-converting enzyme*) led to identify compound **40** (l-*altro*) with a nanomolar activity for all the tested enzymes. In a further work [103], a SAR analysis on compound **40** has been carried out, changing the stereochemistry configuration of azapyranose unit and the type of aromatic ring (Series 2 and Series 3, Figure 11). All these compounds were tested on MMP-1, -3, -9, and TACE and the phenoxyphenyl derivative **46** (l-*ido*) was selected as the best one with an inhibitory activity in the low nanomolar range (*K*_i_ MMP-1: 8.0 nM; *K*_i_ MMP-3: 0.5 nM; *K*_i_ MMP-9: 0.06 nM; *K*_i_ TACE: 2.3 nM). Compound **40** was used as starting point to develop the fourth series [104] of azasugar MMP/ADAM inhibitors, changing its stereochemistry (compound **49**, l-*gulo*) and analyzing different groups in the P1’ position (**50–53**, l-*gulo*). The phenoxyphenyl derivatives **46** and **49** presented an excellent stability in aqueous solution and the best inhibitory activity for MMP-1, MMP-3, MMP-9, and TACE (*K*_i_ in the low nanomolar range). Moreover, azasugar derivative **49** was effective on a mouse TPA-induced epidermal hyperplasia model used to evaluate its efficacy as antipsoriatic agent. The binding mode of compound **49** in MMP-3 catalytic domain determined by crystallographic analysis showed that the stereochemistry of C-2 hydroxyl group was not fundamental for the interaction with the enzyme.

Compound **49** was also tested by other research groups. In 2009, Chikaraishi et al. [105] studied the antiangiogenic activity of **49** by analyzing its suppressing activity against vascular endothelial cell tube formation by an in vitro HUVEC and fibroblast coculture assay and by in vivo retinal neovascularization in a murine ischemia-induced proliferative retinopathy model. In the in vitro angiogenesis model, **49** was able to decrease VEGF-induced HUVEC tube formation. Furthermore, in the in vivo angiogenesis model, administration of **49** reduced retinal neovascularization avoiding side effects on physiological revascularization to the oxygen-induced obliteration area.

In 2018, Sylte et al. [106] reported a study on the binding strengths of compound **49** (Figure 11) and Galardin (Figure 4) for the bacterial MMPs, thermolysin, pseudolysin, and auerolysin, compared to human MMP-9 and MMP-14. The obtained *K*_i_ values of **49** on MMP-9 were approximately 10-fold lower than the previously reported ones. Enzymatic data revealed a stronger inhibition of MMP-9 and MMP-14 by azasugar **49** compared to Galardin probably due to a more favorable interaction of diphenyl ether moiety with the S1’ pocket, as confirmed by a docking study. The opposite effect was found on bacterial MMPs, where Galardin was highly effective while compound **49** was inactive.

**Figure 12:**
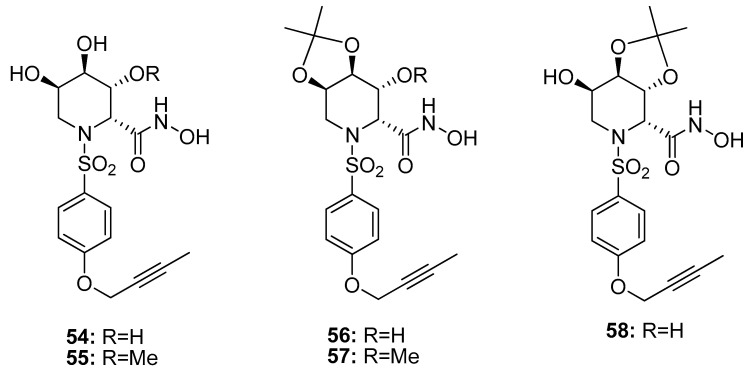
Chemical structure of azasugar-based TACE inhibitors (**54–58**).

The azasugar-based MMP/ADAM inhibitors described so far showed nanomolar inhibition of MMPs and ADAMs but none of these structures presented a good selectivity profile. Based on the SAR analysis of the previous papers, a TACE selective azasugar-based inhibitor series was reported (**54–58**, Figure 12) [107]. In this series the iminosugar stereochemistry was analogous to the one of compounds **49–53** (l-*gulo*) because this was the most suitable in order to inhibit TACE, as described in Moryiama’s papers [102,103,104]. The azasugar was combined with a butyn-2-yloxy aromatic scaffold reported as suitable P1’ substituent in order to achieve TACE selectivity. The new inhibitors were tested by enzymatic assay on human MMPs and TACE, revealing excellent activity for TACE (*K*_i_ 0.53#x2013;1.85 nM), high selectivity over MMP-1 and moderate selectivity over MMP-3 and MMP-9. In particular, compound **56** with a 2,3-*O*-acetonide group had a very potent TACE inhibitory activity (*K*_i_ 0.57 nM) and a good selectivity profile with a 136-fold selectivity for TACE over MMP-9, 52-fold over MMP-3, and 1500-fold over MMP-1.

Biological results for azasugar-based metzincin inhibitors are summarized in Table 2.

## 3. Conclusions

MMPs and ADAMs are zinc-dependent endopeptidases belonging to a larger family of proteases known as metzincins. Upregulation of metzincin activity is a major feature in many serious pathologies such as cancer, inflammatory disorders, neurological diseases, and infections. Several molecules have been discovered in the last years as MMP or ADAM inhibitors presenting very high activity in vitro but also inadequate selectivity for the target enzyme, poor water solubility, and long-term toxicity. For this reason, over the last 15 years, a novel approach to improve solubility and bioavailability of metzincin inhibitors has been the synthesis of carbohydrate-based inhibitors. This strategy consists of the conjugation of a hydrophilic carbohydrate moiety to an aromatic backbone containing the ZBG. In this review we described all the carbohydrate-based metzincin inhibitors reported in literature, classifying them on the basis of the carbohydrate (sugar-based and azasugar-based) and on the basis of the biological target (MMPs or ADAMs). Some promising molecules showing a nanomolar activity and a good selectivity profile for the target enzyme together with an improved water solubility have been discovered. Among sugar-based inhibitors, relevant inhibitory results have been achieved by the GlcNAc-derivative **15**, showing a nanomolar activity for MMP-12, an interesting specificity as well as a good water solubility. Among azasugar-based inhibitors, the phenoxyphenyl derivative **49** is one of the most promising compounds, with high activity and selectivity for MMP-1, MMP-3, and MMP-9 and good inhibitory results in different biological models used to evaluate its antiangiogenic activity. On the basis of the published results here presented, carbohydrate-based inhibitors resulted an emerging class of potent, selective, and water-soluble metzincin inhibitors. Further in vitro and in vivo studies should be performed to finally prove the potentialities of this class of compounds.

## Figures and Tables

**Table 1:** Inhibitory activity (IC_50_ nM) of sugar-based MMP inhibitors against MMP-1, -2, -9, -12.

Compound	MMP-1	MMP-2	MMP-9	MMP-12	Ref
**1**	- ^a^	-	-	490,000	[72]
**2**	-	-	-	790,000	[72]
**3**	286	-	-	14.3	[73]
**4**	-	-	80 (*K*_i_)	17 (*K*_i_)	[74]
**5**	-	30	43	-	[75]
**6**	-	0.2	0.6	0.5	[75]
**8**	-	-	6800	63	[76]
**9**	-	-	9400	73	[76]
**10**	-	-	4400	72	[76]
**11**	-	-	3100	53	[76]
**12**	-	-	1220	36	[76]
**13**	-	-	1200	28	[76]
**14**	19,000	330	1200	18	[76]
**15**	40,000	320	5400	40	[76]
**16**	50,000	100	860	12	[76]
**17**	-	-	1470	42	[76]
**18**	-	-	880	19	[77]
**19**	-	-	1120	98	[77]
**20**	-	-	520	65	[77]
**21**	-	-	1200	18	[77]
**22**	-	-	>50,000	1300	[77]
**23**	-	-	1235	83	[77]
**24**	-	-	890	41	[77]
**25**	-	-	2200	51	[77]

^a^ “-”: not tested or unknown from the corresponding original reference.

**Table 2:** Inhibitory activity (IC_50_ nM) of azasugar-based metzincin inhibitors against ADAM17 and MMP-1, -3, -9.

Compound	ADAM17	MMP-1	MMP-3	MMP-9	Ref
**38**	22	554	44	310	[102]
**39**	40	16	3.7	21	[102]
**40**	71	84	1.7	157	[102]
**41**	21	50	50	47	[102]
**42**	12	25	7.7	4.8	[103]
**43**	510	>850	490	780	[103]
**44**	340	450	85	82	[103]
**45**	8.7	>850	42	64	[103]
**46**	2.3	8.0	0.5	0.06	[103]
**47**	1.6	850	2.6	6.1	[103]
**48**	67	100	1.8	0.9	[103]
**49**	6.2	5.3	0.35	0.097	[104]
**50**	15	26	2	2	[104]
**51**	21	162	50	47	[104]
**52**	1.7	>850	2.1	7.4	[104]
**53**	0.53	128	3.3	14	[104]
**54**	0.53	128	3.3	14.2	[107]
**55**	1.85	90	0.43	12	[107]
**56**	0.57	>850	29.6	77	[107]
**57**	0.84	552	11.5	98	[107]
**58**	1.85	>850	58	118	[107]
